# Medical Students’ attitude to the teaching of Medical Humanities

**DOI:** 10.15694/mep.2018.0000088.1

**Published:** 2018-04-19

**Authors:** Alan Schamroth, Joel Schamroth

**Affiliations:** 1 University College London Medical School; 2 North Middlesex University Hospital

**Keywords:** medical humanities, medical ethics, history of medicine

## Abstract

This article was migrated. The article was marked as recommended.

The teaching of medical humanities to medical students has internationally been regarded as valuable in contributing to a well-rounded medical education. Nevertheless, however valuable one believes the teaching of the medical humanities to be, unless the students perceive the teaching to be useful, relevant, important & interesting, it is unlikely that they will derive maximum clinical benefit from the experience. In this small study, 18 randomly selected first clinical year University College London Medical School students were exposed to humanities teaching during the afternoon session of their 9 days of the year spent in the community attached to a General Practitioners clinic. At the end of the year their views & impression of the teaching of poetry, philosophy of science, biomedical ethics, medical history, museum/art gallery visits & film was obtained. Overall, the students were very positive about their experience & rated all programme components very highly.

## Introduction

Teaching the humanities to medical students has been justified as a means of explaining scientific endeavour ( Baum, 2016) and historical perspective ( Kuhn, 1970), developing ethical awareness ( Arawi, 2010; Russell, Searight & Allmayer, 2014), facilitating personal growth, expanding empathy, self-awareness and sensitivity ( Wolters & Wijnen-Meijer, 2012) and helping students to live with ambivalence & uncertainty ( Klugman, Peel & Beckmann-Mendez, 2011). Art appreciation training has been shown to increase observational & communication skills ( Shapiro, Rucker & Beck, 2006), art ‘practice’ to improve hand-eye coordination and emotional awareness ( Potash, Chen, Lam C et al, 2014) and creative writing to improve narrative competence. Above all, teaching the humanities to medical students has been justified as a counter to the relentless reductionism of medicine. But whatever the intrinsic merit & value of teaching the humanities, unless the students appreciate the experience they are unlikely to obtain the maximum educational potential from it, however well-intentioned. The aim of this study was to evaluate the attitude & perceptions of first clinical year medical students as an important gauge to the acceptability of this humanities programme.

## Educational Initiative

A non-selected group of 18 medical students starting their 1st clinical year in Sept 2016 at University College London Medical School were exposed during the “Medicine in the Community” programme (where students spend one day per month attached to a general practitioner clinic) to afternoon sessions of poetry, history of medicine, ethics & film, philosophy of science and visits to museums and art galleries. To ensure these 18 students did not miss out on any clinical experience compared to their peers they had an intense and concentrated morning session covering the core clinical curriculum (history & examination skills training and clinical tutorials).

The aim of this humanities programme was to provide a scientific, historical, ethical & cultural context to the students’ medical education.

The poetry discussion was facilitated in groups of 6 with the students bringing their own choice of poems related to pre-arranged themes (the life cycle, relationships & illness). They were encouraged to visualise & feel the emotion of the poem and try and link it to hospital patients they had seen in the previous 4 weeks.

The history of medicine was taught as a series of tutorials covering health in ancient hunter-gatherer societies, illness development with the agricultural revolution, health in the ancient world of Egypt, Greece & Rome, in medieval societies though to The Enlightenment and scientific revolution and finally to the modern medicine of today.

The visits to museums and galleries focussed on illustrating medical history, medical anthropology, and illustrations of illness. The museums visited included The British Museum, Wellcome Collection, Science Museum, National Gallery, Museum of the Royal College of Physicians, Hunterian Museum at Royal College of Surgeons, Museum of the Royal College of Obstetrics & Gynaecology, Museum of Apothecaries, Archives of the London School of Hygiene and Tropical Medicine, the Old Operating Theatre, St John’s, Alexander Fleming and Florence Nightingale Museum.

The ethics & law teaching were sessions focussed around films illustrating issues of autonomy, confidentiality, consent, truth-telling, resource allocation and justice. The students were encouraged to explore moral ambiguity and learn approaches to tolerating uncertainty.

Ethics approval was obtained from the University College London Ethics Committee on the 20 ^th^ December 2017 - Project ID 12399/001.

## Evaluation

At the end of the students’ first clinical year (June 2017) the 18 students were asked to anonymously evaluate the different components of this humanities programme in terms of perceived usefulness and interest on a scale of 1-10 (with 1 being poor and 10 being good). The students were also asked to give qualitative feedback with a series of open-ended questionnaires. The numerical data was analysed using descriptive statistics and the qualitative data was analysed using a simple thematic analysis. The response rate was 100%.

Museum visits ranked highest with average students rating of 8.5 (range of 7-10). Students described the visits as ‘great, relaxing, fascinating, enjoyable, stimulating, a wonderful surprise and a treat’. Many students commented that the visits were a welcome break from the stress of the teaching hospitals and realised that learning could be fun. Others said it helped put learning into perspective, broadened their medical horizon, increased their knowledge of medicine & made them more rounded.

The Medical History teaching received an average rating of 8.1 (range 6-10) for usefulness and interest. The students described the tutorials as “enjoyable, fantastic, important, amazing”. Comments included “gave insight into origins of medicine, helpful sense of perspective & understanding of how medicine got to this point, appreciated medicine more for knowing the history”.

The Ethics teaching was equally highly rated at 8.1 (range 7-10). The students described it as “enjoyable, thought-provoking, holistic, useful & informative”. Their comments included “it was a good way to approach complex topics without obvious solutions, loved the discussions & wanted more”. Others said there was no other formal opportunity to discuss these issues elsewhere in the first clinical year & it was particularly valuable in the first few months when they were exposed to the anxieties and stresses of meeting real patients for the first time.

The Poetry session was rated at 8 (range 6-10). The majority of the students admitted that they felt unfamiliar and awkward with this medium initially, but as the year progressed they grew to really appreciate it and spent increasing amounts of time researching and looking for meaningful poems to bring to the sessions. They described the poetry sessions as ‘inspiring, infectious, stimulating & broadened horizons’. Many said that it allowed them to express their own emotions, that it was an excellent way to discover the impact of illness on patients’ lives, that they saw clinical ward situations in a new light and one student said it made them more empathic.

## Conclusion

As the trajectory of medical intervention increasingly concentrates on the micro picture of cells, molecules, and genes, an important concern for medical educators must be that the profession should not lose sight of the whole person, family & community. The medical students come into medicine full of compassion & enthusiasm and the challenge is how to prevent the depersonalising reductionist reality of modern medicine from crushing their idealism and empathy ( Hojat, Vergare, Maxwell et al, 2009).

We believe that teaching the humanities alongside clinical medicine is fundamentally important in equipping the students to become better clinicians by understanding the philosophy underlying the science and also appreciating the moral, social & historic underpinning of the profession. But above all, teaching the humanities may well prevent students becoming demoralised, cynical and disillusioned and equip them to be more resilient ( Macpherson, Hart, Heaver, 2016).

With increasing evidence for this viewpoint, one might ask why the humanities are not taught to medical students in their first clinical year at UCLMS. One of many reasons we suspect could be that the medical schools’ fear this teaching is not acceptable to the students themselves with an eye to the students as discerning consumers.

What this small study suggests is that students not only find the teaching of humanities to be relevant, important & useful, but stimulating & fun. It shows that the students have the maturity and sensitivity to appreciate the benefits of this teaching even if at first they are unfamiliar with the genres (e.g. poetry) & they have the patience & open-mindedness to experience the humanities without initial prejudice before passing their final approving judgement.

The authors hypothesise that the overwhelmingly positive student feedback to the teaching of the medical humanities in this study was influenced by the integrating of the teaching of the humanities into the students clinical day led by the clinicians themselves. We believe this integration of medical science and humanities teaching gave the humanities greater legitimacy & importance in the students view. The students observe their clinical tutors consult with patients, teach clinical skills & give tutorials in the morning and then be equally knowledgeable & at ease discussing ethics, science and medical history in the afternoon.

We further suspect that the positive student response may be influenced by small group teaching. The relevance, immediacy, intimacy, and intensity of the humanities teaching may be diminished if this learning was experienced by the students in a large group format.

Finally, we hypothesise that the student feedback was positively influenced by the continuity of the teaching over the year and the development of the student/clinician-teacher relationship.

An obvious limitation to interpretation was the small size of the study, which would need to be scaled up for more accurate results. Similarly, the final three conjectures possibly influencing the students’ attitude to humanities teaching would also need to be formally tested. Nevertheless, this small study suggests that integrating the teaching of the humanities throughout the first clinical year could be achieved without significant resource implications or curriculum overload and with the endorsement of medical students.

## Notes On Contributors

Alan Schamroth is a Clinical Tutor at UCL Medical School & General Practitioner based in North London.

Joel Schamroth is a junior doctor specialising in acute & general medicine in London.
