# Supplementary material for: Medical Students’ attitude to the teaching of Medical Humanities
Source: MedEdPublish (2016). 2018 Apr 19;7:88. [Version 1] doi: 10.15694/mep.2018.0000088.1 (PMC10711979; doi:10.15694/mep.2018.0000088.1)
Supplement: Peer review history

# Reviewer report: Reviewer response for version 1

**DOI:** 10.21956/mep.19556.r28990

**Published:** 2018-07-01

**Authors:** Irina Markovina

**Affiliations:** 1 Sechenov First Moscow State Medical University (Sechenov University)

This review has been migrated. The reviewer awarded 4 stars out of 5 I find the theme and the idea of this research really interesting and important. Nowadays there is growing understanding that humanities are the necessity in medical education. There is a wide range of subjects and a variety of modes of incorporating humanities in the medical curriculum. What I find especially interesting in this research is that the authors obtained the feedback from the courses participants and analyzed the impressions and opinions upon the completion of the training. It is important to emphasize that it is the evaluation of the impact of the training in Medical Humanities that is the most difficult part of that kind of research. An important continuation of this pilot research would be to find out how the students’ practical attitude and professional behavior change after taking the courses in Medical Humanities. As for the structure and content of the article itself, I would not recommend – if I may - to refer to other authors again in the Conclusion section as these references typically look more appropriate in the Introduction (or sometimes, Discussion) section. The Conclusion section would be more convincing if it focused on the authors’ ideas and comments of on the results obtained.

Reviewer Expertise:

NA

No decision status is available

# Reviewer report: Reviewer response for version 1

**DOI:** 10.21956/mep.19556.r28991

**Published:** 2018-05-15

**Authors:** Susan Van Schalkwyk

**Affiliations:** 1 Stellenbosch University

This review has been migrated. The reviewer awarded 3 stars out of 5 There is a growing focus on incorporating exposure to the humanities as part of the training of health professions students and this article is therefore current and potentially of interest to others in the field. It is evident from the results of the small scale study that the students enjoyed the experience and that as a pilot it opens up a number of possibilities for further exploration. I would have liked to have been given a little more information about how the content and foci were selected, particularly what informed their selection. Were the students given guidelines as to what to look out for during the museum visits, for example, and how were these debriefed? Why were films chosen specifically as the medium for addressing ethics, and so forth? It seems from the reporting that there were no negative comments – was this the case? Did you consider that the absence of assessment (thus the absence of ‘high stakes’) might have contributed to students’ enjoyment? I noted at the end of the article that the clinicians who taught the students in the morning, were also involved in the afternoon. This is a key issue that could have been noted earlier and I would agree that this interaction could have greatly contributed to the success of the intervention.

Reviewer Expertise:

NA

No decision status is available

# Reviewer report: Reviewer response for version 1

**DOI:** 10.21956/mep.19556.r28989

**Published:** 2018-05-09

**Authors:** P Ravi Shankar

**Affiliations:** 1 American International Medical University

This review has been migrated. The reviewer awarded 4 stars out of 5 As an educator involved in teaching-learning of the medical humanities at different medical schools in both Nepal and the Caribbean I found this paper especially interesting. I and my fellow facilitators had ‘introduced’ medical humanities sessions at each of these schools and we could have done so only with the support of the senior academic leadership. Medical humanities (MH) learning is not very common either in Nepal or the Caribbean. At our present institution we offer the module to both medical and nursing students creating opportunities for interprofessional learning. We obtained student feedback about the MH modules we had facilitated and student response seems to be positive. The authors of the paper were lucky to have access to a wide range of museums in a major metropolitan center. We have not used museum visits in our modules mainly because of a shortage of museums both in Nepal and the islands. We also face logistic challenges. This is a well-written manuscript. The questions used for qualitative feedback can be of interest to readers and could be provided. The sessions being facilitated by clinician teachers and being closely linked with the students’ clinical experiences were a major advantage as identified by the authors. In the United Kingdom and developed nations MH is common in medical schools but in many developing regions, learning of MH is not very common and educators face numerous challenges. Recently a number of initiatives in MH have been conducted in the developing world. The references cited are mainly from developed nations and the authors could also describe programs from developing nations.

Reviewer Expertise:

NA

No decision status is available

# Reviewer report: Reviewer response for version 1

**DOI:** 10.21956/mep.19556.r28992

**Published:** 2018-05-04

**Authors:** Subha Ramani

**Affiliations:** 1 Harvard Medical School, Brigham and Women's Hospital

This review has been migrated. The reviewer awarded 3 stars out of 5 This paper addresses an important topic in medical education. It is well written and well referenced and the authors make a strong case for why this problem is important to address. Using mixed method is a good methodological approach.As the authors state, the small numbers of participants make it difficult to draw sweeping conclusions.Additionally, the evaluation of the program targeted mostly satisfaction, the lowest level of the Kirkpatrick pyramid.Nevertheless, it is a solid pilot and I hope the authors study this topic further and provide us with more insights into how humanities influence clinical practice.All medical educators should find aspects of this study useful.I enjoyed reading the paper

Reviewer Expertise:

NA

No decision status is available

# Reviewer report: Reviewer response for version 1

**DOI:** 10.21956/mep.19556.r28988

**Published:** 2018-04-25

**Authors:** Tripti Srivastava

**Affiliations:** 1 Datta Meghe Institute of Medical Sciences Deemed University

This review has been migrated. The reviewer awarded 2 stars out of 5 Indeed Medical Humanities is an integral part of Medicine that has gained its due importance in recent past. The authors have explored the qualitative picture regarding its acceptability among the most important stakeholders i.e the learners. The manuscript clearly depicts their positive reaction towards various aspects of the course. The thematic segregation of qualitative data with an in- depth interpretation regarding the dominant themes for scientific, historical, ethical & cultural context of Medical humanities will be more helpful. Also, the authors can consider a larger sample size, designing a framework for handling 'Medical Humanities' within the curriculum and addressing feasibility issues.

Reviewer Expertise:

NA

No decision status is available
